# Comparative Analysis of Gut Microbiome Diversity, Stability, and Predicted Function in Captive Guanacos (*Lama guanicoe*) and Alpacas (*Vicugna pacos*)

**DOI:** 10.3390/microorganisms14061325

**Published:** 2026-06-13

**Authors:** Yuhong Zhang, Jiawei Zhu, Hufeng Xu, A La Teng Zhu La, Bo Liu, Zichen Zhang, Leshu Liu, Yun Bian, Shun Liang, Mingze Li, Guangrui Zhao, Yanyuan Qiao, Zhanhe Zhang, Ming Xu, Donglin Wu

**Affiliations:** 1 Ordos Longsheng Wildlife Park Co., Ltd., Ordos 017099, China; 15934952955@163.com (Y.Z.); 18347709276@163.com (H.X.); 15924577077@163.com (B.L.); 18347728884@163.com (Z.Z.); 15047348232@163.com (L.L.); 15049408096@163.com (Y.B.); 15147747790@163.com (S.L.); 15044889169@163.com (M.L.); 2 College of Animal Science, Inner Mongolia Agricultural University, Hohhot 010018, China; zjw1911024745@emails.imau.edu.cn (J.Z.); alatengzhula@imau.edu.cn (A.L.T.Z.L.); dk506@emails.imau.edu.cn (G.Z.); 15049394415@163.com (Y.Q.); 506@emails.imau.edu.cn (Z.Z.); ndxm@imau.edu.cn (M.X.)

**Keywords:** gut microbiota, guanaco, alpaca, community stability, co-occurrence network, domestication, 16S rRNA

## Abstract

The gut microbiota plays a vital role in host health. In response to the scarcity of comparative studies examining wild and domesticated South American camelids under identical captive conditions, this study was conducted to compare the gut microbiota of 16 captive guanacos (*Lama guanicoe*) and 8 alpacas (*Vicugna pacos*) housed in the same zoo and fed identical diets, using 16S rRNA gene sequencing and multiple ecological metrics for analysis. Alpha diversity indices (Shannon, observed richness, and Shannoneven) did not differ between the two species, but beta diversity (principal component analysis) indicated significant separation (*p* < 0.05), and the guanacos exhibited significantly lower within-group Bray–Curtis dissimilarity, indicating more consistent microbial communities. Guanacos exhibited a lower average variation degree (AVD), indicating greater community stability, a broader niche, and a co-occurrence network with 81.1% positive edges and high modularity (0.691). In contrast, the alpacas showed a higher AVD (lower stability), a narrower niche, and a network with only 62.2% positive edges and lower modularity (0.534). Linear discriminant analysis effect size analysis revealed that *Monoglobus* and *Bacteroides* are enriched in guanacos, while *Rikenellaceae_RC9_gut_group* is enriched in alpacas. Functional predictions revealed that alpacas had higher predicted abundances of potentially pathogenic taxa and Kyoto Encyclopedia of Genes and Genomes pathways related to *Staphylococcus aureus* infection (*p* < 0.05). These findings demonstrate that, despite sharing environments, guanacos have a more stable, generalist-dominated gut microbiota with a higher proportion of positive co-occurrences, whereas alpacas exhibit a less stable, specialist-oriented community with a higher proportion of negative co-occurrences and greater predicted pathogenic potential. These results suggest that domestication may have contributed to the observed divergence in gut microbial ecology between the two species.

## 1. Introduction

The gut microbiota plays an essential role in host nutrition, immune function, and resistance to pathogens [1]. In herbivorous mammals, the microbial fermentation of plant fiber is critical for energy acquisition. South American camelids—guanacos (*Lama guanicoe*; GAS) and alpacas (*Vicugna pacos*; AL)—are pseudo-ruminants that depend on a complex gut microbiota to digest fibrous forages [2]. Guanacos are wild or semi-wild camelids inhabiting diverse environments ranging from the Andes to Patagonia, whereas alpacas are domesticated descendants of the vicuña, selectively bred for fiber (wool) production over millennia [3]. Both species hold ecological and economic importance, yet their gut microbiomes have only rarely been compared systematically.

Host genetics, diet, and environment collectively shape the gut microbiota [4,5]. Under captive conditions where diet and housing are standardized, interspecific differences are more likely attributable to host-specific factors such as evolutionary history and physiology [6]. Although several studies have characterized the fecal microbiota of alpacas, most have focused on single-species inventories or disease states (e.g., neonatal diarrhea) [7,8], and no comprehensive comparison with guanacos has been reported. Furthermore, the effects of domestication on the gut microbiota remain debated: some studies in pigs and other mammals have found that domestication reduces beneficial bacteria and increases opportunistic pathogens [9], while others report higher alpha diversity in domesticated populations [10]. The guanaco–alpaca pair offers a unique model to address this controversy, as the two species share a recent common ancestor but have diverged under natural versus human-directed selection.

All animals in this study were housed in the same zoo with the same diet and environment, minimizing external confounding factors. Few studies have compared the gut microbiota of guanacos and alpacas using integrated ecological analyses. We therefore examined microbial diversity, stability, community structure, and functional potential. We hypothesized that domestication may contribute to significant differences in gut microbial diversity, stability, and functional potential between guanacos and alpacas. This study provides a microbiome reference for guanaco conservation and alpaca health management and improves our understanding of how domestication shapes gut microbial ecology.

## 2. Materials and Methods

### 2.1. Animals and Sample Collection

All animal procedures were approved by the Institutional Animal Care and Use Committee of Inner Mongolia Agricultural University (protocol No. NND2025019) and were carried out in accordance with relevant guidelines and regulations.

This study was conducted at Ordos Longsheng Wildlife Park (Ordos, China; 39.84° N, 109.89° E) in June 2025. The region has a mid-temperate continental monsoon climate; during the sampling period, the average ambient temperature was 15.1 ± 8.3 °C and the average relative humidity was 32.1 ± 15.9% (mean ± SD). A total of 24 adult individuals were included: 16 captive guanacos (*Lama guanicoe*; 10 females and 6 males aged 3–5 years with a body weight of 90.5 ± 4.7 kg) and 8 alpacas (*Vicugna pacos*; 6 females and 2 males aged 3–5 years with a body weight of 65.6 ± 2.9 kg). All animals were group-housed in enclosures that provided both indoor and outdoor exercise areas with dry, clean bedding. All animals were confirmed to be healthy by a resident veterinarian based on a physical examination, normal behavior, and the absence of diarrhea or other clinical signs for at least two weeks prior to sampling.

The animals had ad libitum access to fresh water and were fed the same batch of alfalfa hay twice daily. They also received a commercial pelleted concentrate (200 g/animal/day) and had free access to a salt-mineral block. The salt-mineral block contained 8.66% sodium chloride (measured by the supplier). Representative samples of the hay and pelleted feed were analyzed for their nutritional composition using near-infrared reflectance spectroscopy (NIRS; FOSS NIRS^TM^ DS 2500, Hillerød, Denmark). The main nutritional components of hay were as follows: dry matter (DM), 91.1%; crude protein (CP), 18.6%; acid detergent fiber (ADF), 31.4%; neutral detergent fiber (NDF), 38.98%; crude fat, 2.8%; ash, 9.27%; calcium, 1.31%; phosphorus, 0.28%. The main nutritional components of the pelleted feed were as follows: DM, 89.2%; CP, 18.0%; ADF, 19.7%; NDF, 26.0%; crude fat, 4.9%; ash, 7.16%; calcium, 1.12%; phosphorus, 0.56%.

On the day of sampling, each animal was gently guided into a single-animal restraint passage (allowing the passage of only one animal at a time) to minimize stress. Fecal samples were collected by rectal stimulation: a sterile, disposable glove was worn, and the index and middle fingers were lubricated with sterile saline and then gently inserted into the rectum to stimulate defecation. Immediately after expulsion, the fecal bolus was homogenized using a sterile spatula, and approximately 2 g of the homogenized sample was transferred to a sterile, enzyme-free cryogenic vial. The vial was snap-frozen in liquid nitrogen within 5 min of collection and then transported to the laboratory and stored at −80 °C until DNA extraction. All sampling procedures were performed between 8:00 and 10:00 a.m. to control for circadian variation. To prevent cross-contamination, gloves were changed between animals, and all instruments were cleaned with 70% ethanol.

### 2.2. Sample Analysis and Data Analysis

#### 2.2.1. DNA Extraction and PCR Amplification

Total microbial genomic DNA was extracted from each intestinal content sample using the E.Z.N.A.^®^ soil DNA Kit (Omega Bio-tek, Norcross, GA, USA), strictly following the manufacturer’s protocol. DNA integrity and concentration were assessed via 1.0% agarose gel electrophoresis and using a NanoDrop™ 2000 spectrophotometer (Thermo Fisher Scientific, Waltham, MA, USA). Extracted DNA was stored at −80 °C until further use.

The V3–V4 hypervariable region of the bacterial 16S rRNA gene was amplified using the primer pairs 338F (5′-ACTCCTACGGGAGGCAGCAG-3′) and 806R (5′-GGACTACHVGGGTWTCTAAT-3′) [11] on a T100 Thermal Cycler PCR thermocycler (BIO-RAD, Hercules, CA, USA). Each 20 µL PCR mixture contained 4 µL of 5× FastPfu buffer, 2 µL of 2.5 mM dNTPs, 0.8 µL of each primer (5 µM), 0.4 µL of FastPfu polymerase, approximately 10 ng of template DNA, and nuclease-free water to volume. The thermal cycling conditions consisted of initial denaturation at 95 °C for 3 min, followed by 27 cycles of denaturation at 95 °C for 30 s, annealing at 55 °C for 30 s, and extension at 72 °C for 45 s, with a final extension step at 72 °C for 10 min. The reaction was then held at 4 °C. Amplicons were separated on 2% agarose gel, excised, and purified using a PCR Clean-Up Kit (YuHua, Shanghai, China) according to the manufacturer’s instructions. Purified products were quantified with a Qubit™ 4.0 fluorometer (Thermo Fisher Scientific, USA).

Equimolar amounts of purified amplicons were pooled and subjected to paired-end sequencing on an Illumina NextSeq™ 2000 platform (Illumina, San Diego, CA, USA), following the standard protocols provided by Majorbio Bio-Pharm Technology Co., Ltd. (Shanghai, China). The raw sequence reads have been deposited in the NCBI Sequence Read Archive (SRA) under accession number PRJNA1458602.

#### 2.2.2. Sequence Processing, Amplicon Sequence Variants (ASVs) Inference, and Downstream Analysis

**Figure 1:**
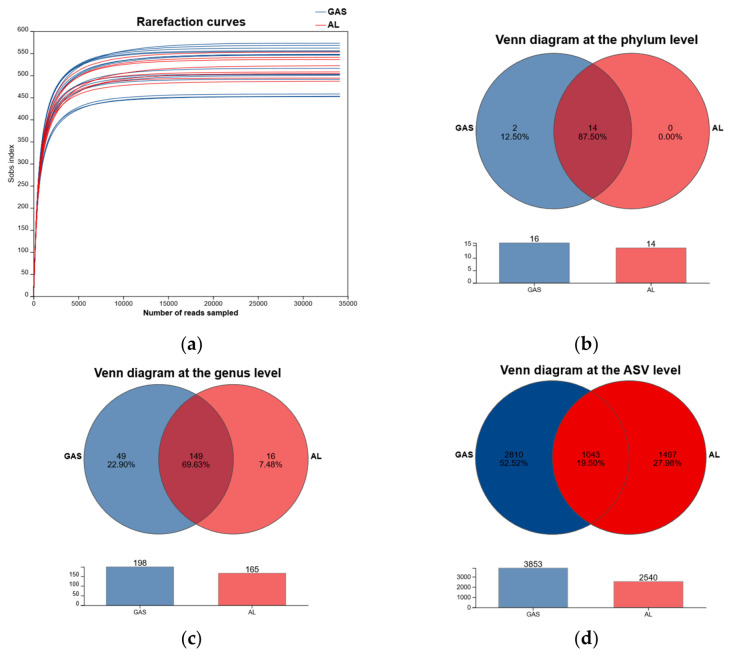
Rarefaction curves and Venn diagrams of gut microbiota in guanacos (GAS) and alpacas (AL). (**a**) Rarefaction curves for the sequencing of the 24 intestinal content samples; the curves plateaued by 36,496 sequences per sample. (**b**) Venn diagram at the phylum level for the two groups. (**c**) Venn diagram at the genus level. (**d**) Venn diagram at the ASV level. Numbers indicate unique and shared taxa. No statistical tests were applied to the Venn diagrams. GAS: *n* = 16; AL: *n* = 8.

Raw demultiplexed reads were quality-filtered using fastp (version 0.23.4) [12] and merged with FLASH (version 1.2.11) [13]. High-quality merged sequences were then denoised with the DADA2 plugin [14], implemented in QIIME 2 (version 2020.2) [15] using default parameters, which generated ASVs at single-nucleotide resolution by modeling error profiles within samples. To minimize bias caused by uneven sequencing depth across samples, the amplicon sequence variant (ASV) abundance table was rarefied to 36,496 sequences per sample. This threshold was selected because it corresponded to the lowest denoised sequence count among the 24 samples after DADA2 processing (sample GAS8; Supplementary Table S1). The rarefaction curves (Figure 1a) reached a stable plateau well before this depth, and the average Good’s coverage was 99.99% at 36,496 sequences, confirming that the sequencing depth was sufficient to capture the majority of microbes in both groups.

Taxonomic assignment of ASVs was performed against the SILVA 16S rRNA database (release 138.2) using the naive Bayes consensus taxonomy classifier available in QIIME 2. The functional potential of the gut metagenome was predicted using PICRUSt2version 2.2.0 (Phylogenetic Investigation of Communities by Reconstruction of Unobserved States) [16] based on the ASV representative sequences. Additionally, microbial phenotypes—including aerobic, anaerobic, mobile element-containing, facultatively anaerobic, biofilm-forming, Gram-negative, Gram-positive, potentially pathogenic, and stress-tolerant microbes—were predicted using the BugBase online tool (https://bugbase.cs.umn.edu/index.html, accessed on 6 April 2026).

### 2.3. Statistical Analysis

All statistical analyses were performed using R version 3.3.1 (subsequent validation with R 4.2.1 confirmed identical results) and the Majorbio Cloud platform (https://cloud.majorbio.com, accessed in 6 April 2026). A two-tailed Wilcoxon rank-sum test was used for all pairwise comparisons between GASs and ALs, unless otherwise specified. Statistical significance was set at *p* < 0.05. Multiple testing correction was applied using the Benjamini–Hochberg false discovery rate (FDR) method, with an adjusted *p*-value threshold of <0.05. Adjusted *p*-values are reported in the Supplementary Tables.

Alpha diversity indices (Shannon, observed richness (Sobs), and Shannoneven) were calculated at the ASV level. Beta diversity was visualized using principal component analysis (PCA), and group differences in community composition were tested via analysis of similarities (ANOSIM) with 999 permutations (vegan package). The ANOSIM R-statistic and its *p*-value are reported. Pairwise Bray–Curtis dissimilarities within each group were calculated at the ASV level.

The average variation degree (AVD) was calculated as a measure of community stability (lower AVD values = higher stability, i.e., less inter-individual variation). Briefly, AVD quantifies the average deviation of ASV relative abundances from the group mean across all ASVs and samples. For the detailed calculation procedure, please refer to Xun et al. (2021) [17]. Because only one AVD value was obtained per group, no inter-group statistical test was performed, and values are reported descriptively. Niche breadth (Bcom) and niche overlap were computed at the ASV level after prevalence filtering (≥0.01) and compared between groups using the Wilcoxon test.

Co-occurrence networks were constructed separately for GASs and ALs using the Wekemo Bioincloud platform (https://www.bioincloud.tech, accessed in 6 April 2026). For each group, the top 50 most abundant genera were used, and pairwise associations were calculated using the propr package. Edges with |propr| > 0.6 and *p* < 0.05 were retained. Topological parameters (nodes, edges, average degree, clustering coefficient, modularity, and number of communities) were computed. For each empirical network, 100 random networks of equal size were generated to test for non-random structure. The proportions of positive versus negative edges were compared between groups using a chi-square test (SPSS v27.0.1).

Differential abundances of phyla and genera were analyzed using the Wilcoxon test. To identify taxa with the greatest discriminatory power, linear discriminant analysis effect size (LEfSe) was performed. The analysis was based on relative abundance data from the phylum to genus levels. The non-parametric Kruskal–Wallis test was first applied to detect significantly different taxa (phylum to genus level), followed by linear discriminant analysis (LDA) to estimate effect sizes. An LDA score threshold > 2.0 was considered significant [18].

Predicted bacterial functions (Kyoto Encyclopedia of Genes and Genomes, KEGG, Level 3 pathways) were obtained using PICRUSt2, and microbial phenotypes were predicted using BugBase. Absolute read counts for KEGG pathways and relative abundances of BugBase phenotypes were compared between groups using the Wilcoxon test.

## 3. Results

### 3.1. Sequencing Information and Microbial Diversity

A total of 1,081,367 clean reads were obtained from the 24 fecal samples (Supplementary Table S1). Per-sample clean read counts ranged from 36,496 to 51,353 (mean ± SD: 45,057 ± 3448), with a mean read length of 411.65 bp. Good’s coverage indices exceeded 99.99% for all samples, indicating comprehensive bacterial identification and sufficient sequencing depth. The rarefaction curves (Figure 1a) plateaued well before 36,496 sequences per sample, confirming that the sequencing depth was adequate to capture the majority of microbes in both groups.

The Venn diagram below (Figure 1b–d) effectively demonstrates the underlying diversity and richness within these microbial communities by showing how many unique ASVs are present in each group. Notably, among those identified, 14 phyla (comprising 87.50%), 149 genera (representing 69.63%), and 1043 ASVs (comprising 19.50%) are common across both groups, revealing a core microbiome component that is consistent within the intestinal environment of these animals. However, beyond this shared microbial baseline at the phylum level, each of the two groups has a distinct set of microbial phyla, with two unique phyla (12.50%) found in the GAS group and zero (0%) in the AL group. At the genus level, 49 unique genera (22.90%) were found in the GAS group and 16 (7.48%) were found in the AL group. Finally, at the ASV level, there were 2810 unique ASVs (52.52%) in the GAS group and 1497 (27.98%) in the AL group. This indicates that, while there is a common core of microbial taxa, each treatment is associated with a distinct microbial signature, with the GAS group standing out for its higher level of specific organisms.

**Figure 2:**
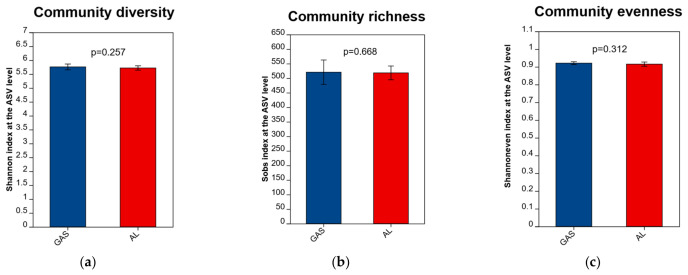

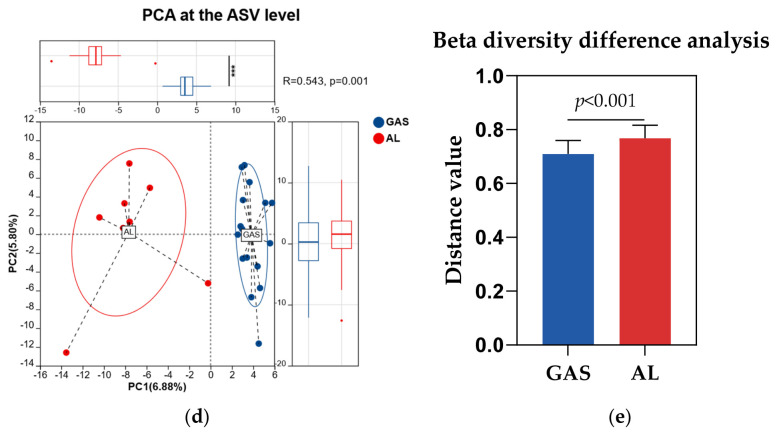
Alpha and beta diversity of the gut microbiota in guanacos (GAS) and alpacas (AL). (**a**) Shannon diversity index (community diversity); error bars represent standard deviation (SD). (**b**) Sobs index (community richness); error bars represent SD. (**c**) Shannoneven index (community evenness); error bars represent SD. (**d**) PCA plot of beta diversity at the ASV level. Differences in community composition were tested via ANOSIM with 999 permutations (R = 0.543, *p* < 0.05). (**e**) Boxplot of pairwise Bray–Curtis dissimilarities within the GAS and AL groups. Lower values indicate greater similarity among individuals within the group. GAS: *n* = 16; AL: *n* = 8. *** *p* ≤ 0.001.

The alpha diversity index results are presented in Figure 2a. No significant differences were observed in any of the alpha diversity indices between the GAS and AL groups. Specifically, the Shannon diversity index (community diversity), Sobs index (ASV-level richness), and Shannoneven index (community evenness) all showed comparable values between the two groups (Wilcoxon rank-sum test, two-tailed, *p* > 0.05 for all indices), as shown in Figure 2a–c.

In contrast, the beta diversity analysis revealed a significant separation in gut microbial community composition between the two groups. The PCA plot based on ASV-level abundance data demonstrated distinct clustering patterns for the GAS and AL samples (Figure 2d). The ANOSIM test (999 permutations) confirmed that the observed between-group differences were statistically significant (R = 0.543, *p* < 0.05), indicating that the gut microbial community structure differs substantially between captive guanacos and alpacas despite their similar alpha diversity profiles.

To further compare inter-individual variability between the two groups, pairwise Bray–Curtis dissimilarities were calculated for all samples in each group. The average within-group dissimilarity was significantly lower in GASs (mean ± SD: 0.709 ± 0.050) than in ALs (0.768 ± 0.048; Wilcoxon rank-sum test, *p* = 3.33 × 10^−7^; Figure 2e), indicating that guanaco individuals harbor more similar gut microbial communities (i.e., lower individual variation) than alpacas.

### 3.2. Gut Microbiota Stability

**Figure 3:**
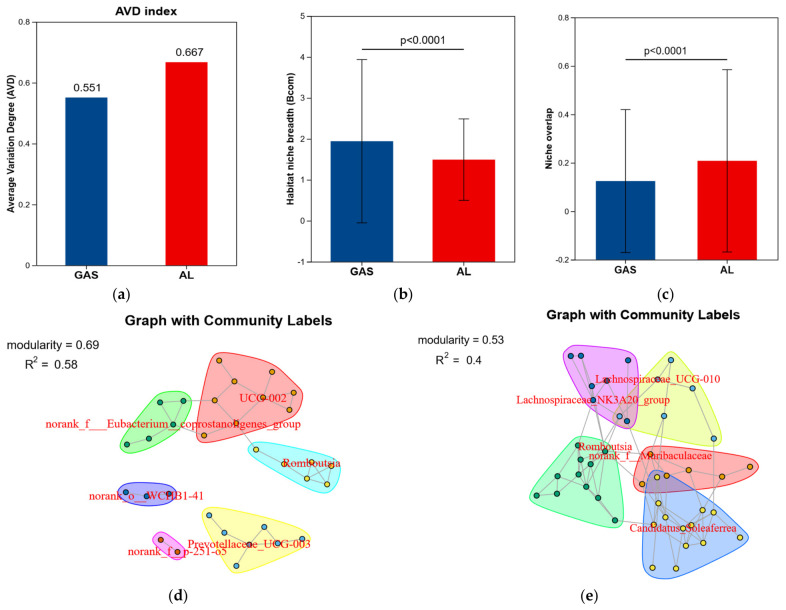
Comparisons of microbial community stability, niche characteristics, and co-occurrence networks in the gut microbiota of captive guanacos (GAS) and alpacas (AL). (**a**) Average variation degree (AVD); lower values indicate higher community stability. Because only one AVD value was obtained per group, no statistical comparison was performed; values are presented descriptively. (**b**) Habitat niche breadth (Bcom) at the ASV level; greater breadth reflects higher metabolic plasticity and a higher proportion of generalist taxa. Error bars represent SD (**c**) Niche overlap index; higher values indicate greater sharing of ecological resources. Error bars represent SD. (**d**) Co-occurrence network of the GAS group. (**e**) Co-occurrence network of the AL group. For (**d**,**e**), nodes represent genera; edges represent significant propr values (|propr| > 0.6, *p* < 0.05). Network statistics are provided in Supplementary Tables S4–S6.

The AVD was calculated to compare gut microbial community stability between the two host species. A lower AVD was observed in the GAS group compared to the AL group, indicating that the gut microbiota of species with a lower AVD exhibits higher community stability (Figure 3a).

Niche breadth analysis revealed a significant difference between the two groups (*p* < 0.05), with GASs displaying a significantly broader habitat niche (Bcom) at the ASV level (Figure 3b). This finding suggests that the gut microbial communities of species with broader niches possess greater metabolic plasticity and a higher proportion of generalist taxa, which may confer reduced environmental sensitivity relative to other species.

Niche overlap also differed significantly between the two groups (*p* < 0.05). ALs exhibited a higher niche overlap index than GASs (Figure 3c), indicating distinct patterns in how the two host species’ gut microbial communities utilize and share environmental resources. The *p*-value (*p*< 0.001) further suggests that the two host species have fundamentally different microbial resource utilization strategies.

To further explore the interaction patterns underlying community stability, co-occurrence network analysis was performed. Group-specific networks were built using the top 50 most abundant genera in each group based on propr values (|propr| > 0.6, *p* < 0.05). The GAS network (Figure 3d) consisted of 32 nodes and 37 edges, of which 30 (81.1%) were positive and 7 (18.9%) were negative (Supplementary Tables S4 and S6). In contrast, the AL network (Figure 3e) contained 43 nodes and 90 edges, with 56 positive (62.2%) and 34 negative (37.8%) correlations (Supplementary Tables S5 and S6). A chi-square test of independence was performed to compare the proportion of positive and negative edges between the GAS and AL networks. The proportion of positive edges was significantly higher in GASs (81.1%) than in ALs (62.2%; χ^2^(1) = 4.266, *p* = 0.039).

As shown in Supplementary Table S6, both networks exhibited modularity values exceeding those of random networks (GASs: 0.691 vs. random 0.521; ALs: 0.534 vs. random 0.385), with GASs showing substantially higher modularity. The predominance of positive associations in GASs (81.1%) suggests a microbial community dominated by positively co-occurring taxa, characteristic of generalist-dominated ecosystems with a broad niche and high stability. The AL network, while still dominated by positive edges (62.2%), contained nearly twice the proportion of negative correlations compared to the GAS network (37.8% vs. 18.9%), which is consistent with a higher degree of negative co-occurrence among its more specialist-like taxa. These network-level differences align with the observed lower AVDs and broader niches in GASs and the higher AVDs and narrower niches in ALs.

### 3.3. Composition and Differential Bacterial Taxonomic Analysis

**Figure 4:**
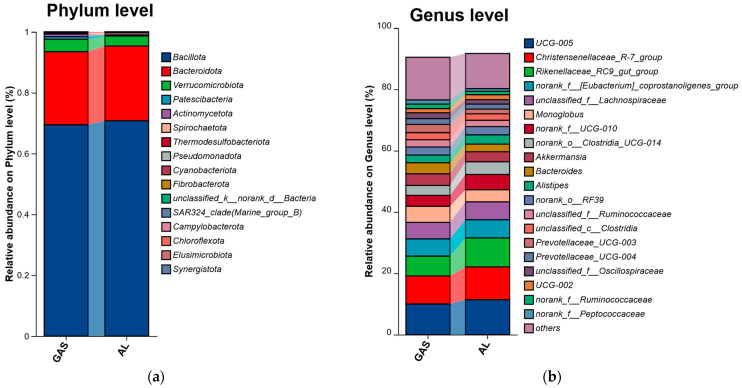

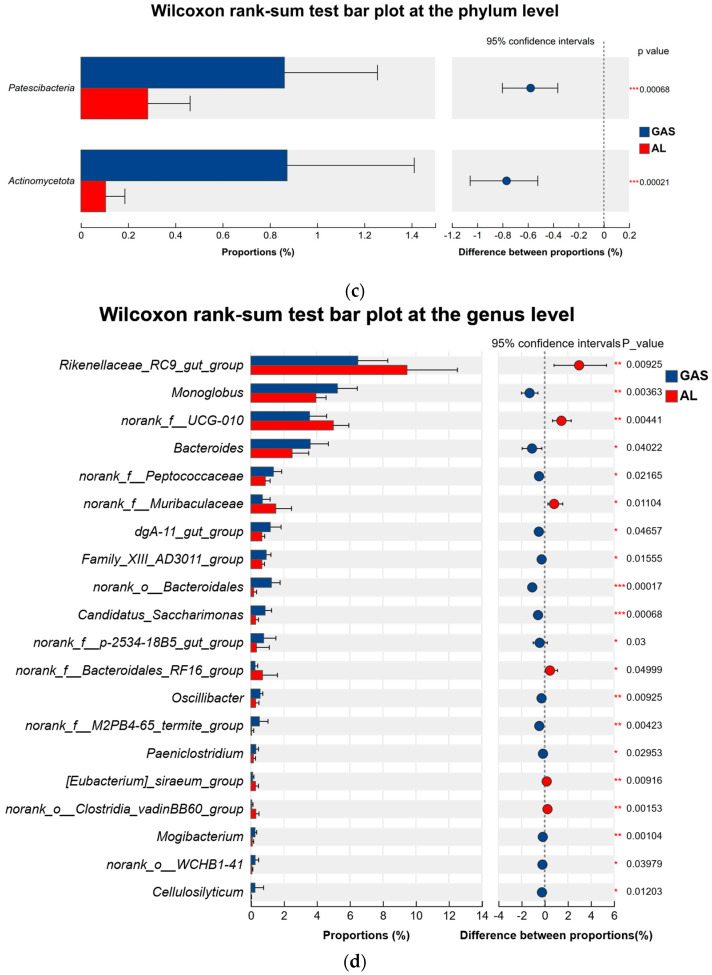
Composition and differential abundance of gut microbiota at phylum and genus levels in guanacos (GAS) and alpacas (AL). (**a**) Community bar diagram at the phylum level. (**b**) Community bar diagram at the genus level (top 20 genera). (**c**) Differentially abundant phyla. Error bars represent SD. (**d**) Differentially abundant genera. Error bars represent SD. 0.01 < * *p* < 0.05, 0.001 < ** *p* ≤ 0.01, *** *p* ≤ 0.001. Complete results are provided in Supplementary Tables S2 (phylum) and S3 (genus).

The top 20 most abundant genera (ranked by mean relative abundance within each group) were described. As shown in Figure 4a, at the phylum level, the gut microbiota of the GAS group was dominated by *Bacillota* (69.46%), *Bacteroidota* (24.02%), and *Verrucomicrobiota* (4.08%). These three phyla collectively accounted for approximately 97.6% of the total microbial community. Other phyla, each with a relative abundance below 1%, included *Patescibacteria* (0.86%), *Actinomycetota* (0.87%), *Spirochaetota* (0.22%), *Thermodesulfobacteriota* (0.29%), *Pseudomonadota* (0.09%), *Cyanobacteriota* (0.04%), *Fibrobacterota* (0.01%), and several minor phyla (each <0.02%).

In the AL group, a similar phylum-level composition was observed. The most abundant phyla were *Bacillota* (70.78%), *Bacteroidota* (24.55%), and *Verrucomicrobiota* (3.31%), together representing approximately 98.6% of the total gut microbiota. The remaining phyla, all with a relative abundance below 1%, included *Spirochaetota* (0.61%), *Patescibacteria* (0.28%), *Thermodesulfobacteriota* (0.16%), *Actinomycetota* (0.10%), *Pseudomonadota* (0.07%), *Fibrobacterota* (0.05%), *Cyanobacteriota* (0.03%), and other less abundant phyla (each <0.02%). Notably, *Elusimicrobiota* and *Synergistota* were detected in the GAS group only, but at very low abundances (<0.002%).

Overall, the gut microbial communities of both GASs and ALs were dominated by the same three major phyla (*Bacillota*, *Bacteroidota*, and *Verrucomicrobiota*), with no substantial differences in their relative abundances between the two host species.

As shown in Figure 4b, at the genus level, the top 20 most abundant taxa in the GAS group were *UCG-005* (10.01%), *Christensenellaceae R-7 group* (9.15%), *Rikenellaceae RC9 gut group* (6.48%), *norank_f__[Eubacterium]_coprostanoligenes_group* (5.61%), *unclassified_f__Lachnospiraceae* (5.39%), *Monoglobus* (5.24%), *norank_f__UCG-010* (3.55%), *norank_o__Clostridia_UCG-014* (3.24%), *Akkermansia* (3.82%), *Bacteroides* (3.59%), *Alistipes* (2.51%), *norank_o__RF39* (2.62%), *unclassified_f__Ruminococcaceae* (2.36%), *unclassified_c__Clostridia* (2.33%), *Prevotellaceae UCG-003* (2.67%), *Prevotellaceae UCG-004* (1.89%), *unclassified_f__Oscillospiraceae* (1.90%), *UCG-002* (1.39%), *norank_f__Ruminococcaceae* (1.46%), and *norank_f__Peptococcaceae* (1.36%). These 20 genera collectively accounted for approximately 72.1% of the total gut microbiota in the GAS group.

In the AL group, the top 20 most abundant genera were *UCG-005* (11.41%), *Christensenellaceae R-7 group* (10.69%), *Rikenellaceae RC9 gut group* (9.46%), *norank_f__[Eubacterium]_coprostanoligenes_group* (5.93%), *unclassified_f__Lachnospiraceae* (5.85%), *norank_f__UCG-010* (5.00%), *norank_o__Clostridia_UCG-014* (4.13%), *Monoglobus* (3.94%), *Akkermansia* (3.25%), *Alistipes* (3.04%), *Bacteroides* (2.50%), *norank_o__RF39* (2.66%), *unclassified_f__Ruminococcaceae* (2.08%), *unclassified_c__Clostridia* (2.09%), *Prevotellaceae UCG-003* (1.55%), *Prevotellaceae UCG-004* (1.63%), *unclassified_f__Oscillospiraceae* (1.44%), *UCG-002* (1.63%), *norank_f__Muribaculaceae* (1.51%), and *UCG-009* (1.12%). These 20 genera represented approximately 73.7% of the total gut microbiota in the AL group.

At the phylum level (Figure 4c), the relative abundances of *Patescibacteria* and *Actinomycetota* were significantly lower in the AL group compared to the GAS group (*p* < 0.05). The results of all other phyla are provided in Supplementary Table S2.

At the genus level, among the top 20 most abundant genera, the AL group exhibited a significantly lower relative abundance of the following taxa compared to the GAS group (Figure 4d): *Monoglobus*, *Bacteroides*, *norank_f__Peptococcaceae*, *dgA-11_gut_group*, *Family_XIII_AD3011_group*, *norank_o__Bacteroidales*, *Candidatus Saccharimonas*, *norank_f__p-2534-18B5_gut_group*, *Oscillibacter*, *norank_f__M2PB4-65_termite_group*, *Paeniclostridium*, *Mogibacterium*, *norank_o__WCHB1-41*, and *Cellulosilyticum* (*p* < 0.05).

Conversely, the AL group showed a significantly higher relative abundance of the following genera within the top 20 compared to the GAS group (Figure 4d): *Rikenellaceae_RC9_gut_group*, *norank_f__UCG-010*, *norank_f__Muribaculaceae*, *norank_f__Bacteroidales_RF16_group*, *[Eubacterium] siraeum group*, and *norank_o__Clostridia_vadinBB60_group* (*p* < 0.05).

The complete list of genera with significant abundance differences between the two groups is provided in Supplementary Table S3.

**Figure 5:**
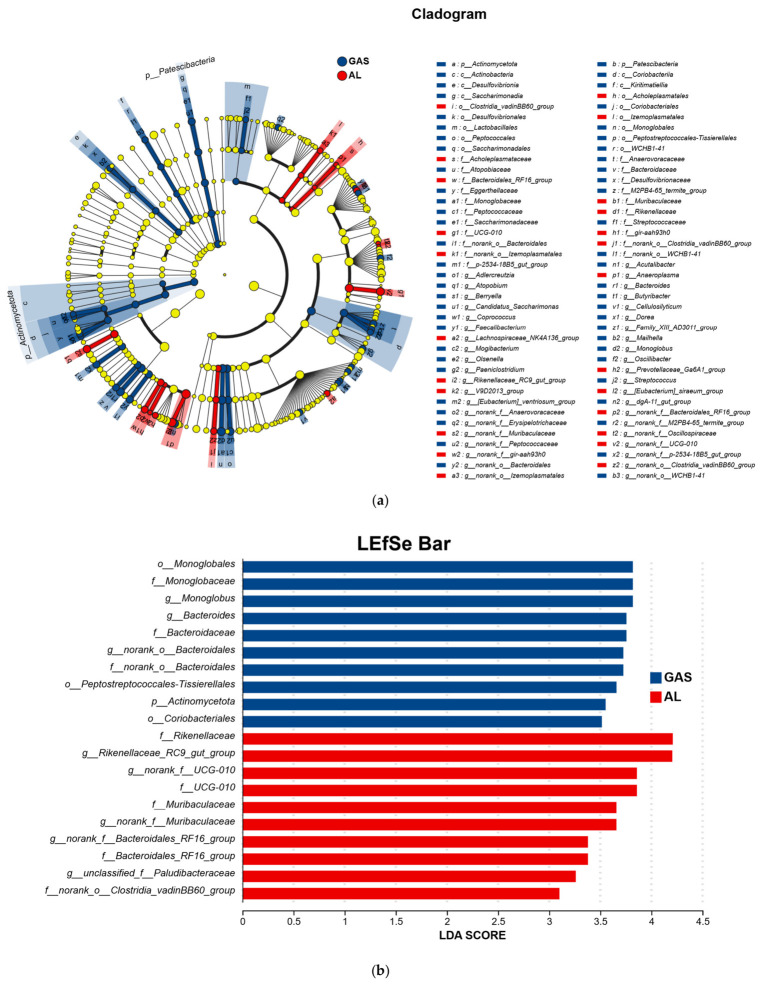
Linear discriminant analysis effect size (LEfSe) analysis of gut microbiota between guanacos (GAS) and alpacas (AL). (**a**) Cladogram illustrating the phylogenetic distribution of differentially abundant taxa from phylum to genus levels. Circles from inside to outside represent phylum to genus levels. Statistical procedure: Kruskal–Wallis test (*p* < 0.05, uncorrected for feature selection) followed by linear discriminant analysis (LDA) to estimate effect size; an LDA threshold > 2.0 was considered significant. Red nodes indicate enrichment in AL; blue nodes in GAS. Yellow circles represent microbial taxa that were not significantly enriched in either group (LDA score ≤ 2.0). A complete list of all 82 discriminative taxa is provided in Supplementary Table S7. A complete list of all 82 discriminative taxa is provided in Supplementary Table S7. (**b**) LDA bar plot showing the top 10 discriminative taxa with the highest LDA scores (LDA > 2.0) in each group. Bar length represents the effect size (LDA score).

To further identify the taxa with the highest discriminatory power between the two host species, LEfSe analysis was performed. A total of 82 taxa showed significant differences in relative abundance between the GAS and AL groups (Supplementary Table S7). The cladogram below (Figure 5a) further illustrates the phylogenetic distribution of these discriminative taxa. In the GAS group, the top 10 taxa with the highest LDA scores were *o__Monoglobales*, *f__Monoglobaceae*, *g__Monoglobus*, *g__Bacteroides*, *f__Bacteroidaceae*, *f__norank_o__Bacteroidales*, *g__norank_o__Bacteroidales*, *o__Peptostreptococcales-Tissierellales*, *p__Actinomycetota*, and *o__Coriobacteriales* (Figure 5b). In contrast, the AL group was characterized by a distinct set of discriminative taxa, including *f__Rikenellaceae*, *g__Rikenellaceae_RC9_gut_group*, *g__norank_f__UCG-010*, *f__UCG-010*, *f__Muribaculaceae*, *g__norank_f__Muribaculaceae*, *g__norank_f__Bacteroidales_RF16_group*, *f__Bacteroidales_RF16_group*, *g__unclassified_f__Paludibacteraceae*, and *f__norank_o__Clostridia_vadinBB60_group*. These results corroborate the differential abundance findings from the Wilcoxon rank-sum tests and highlight key microbial lineages that distinguish the gut microbiota of captive guanacos and alpacas. While the LEfSe analysis identified 82 discriminative taxa (Supplementary Table S7), readers should note that some of these taxa are present at very low relative abundances, and their biological significance requires further validation.

### 3.4. Microbial Function

Functional prediction of the gut bacterial genome was performed using KEGG Level 3 pathways. Absolute read counts were compared between the AL and GAS groups. A total of 21 pathways (a total of 308 pathways were detected) showed statistically significant differences (*p* < 0.05; Supplementary Table S8).

Pathways significantly higher in the AL group than in the GAS group included *Staphylococcus aureus* infection, D-Arginine and D-ornithine metabolism, bladder cancer, degradation of aromatic compounds, caprolactam degradation, naphthalene degradation, thyroid hormone signaling pathway, longevity regulating pathway, cell cycle—yeast, drug metabolism—cytochrome P450, toluene degradation, metabolism of xenobiotics by cytochrome P450, cell cycle, and retinol metabolism (*p* < 0.05). Pathways significantly lower in the AL group than in the GAS group included flavone and flavonol biosynthesis, Shigellosis, platinum drug resistance, PI3K-Akt signaling pathway, glucagon signaling pathway, insulin signaling pathway, and central carbon metabolism in cancer (*p* < 0.05).

**Figure 6:**
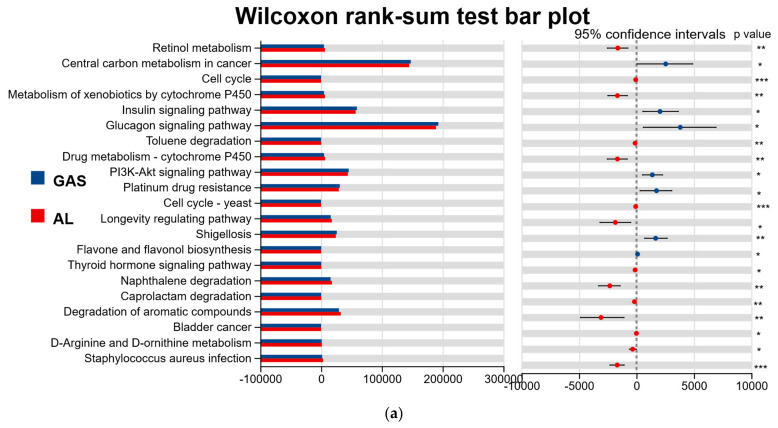

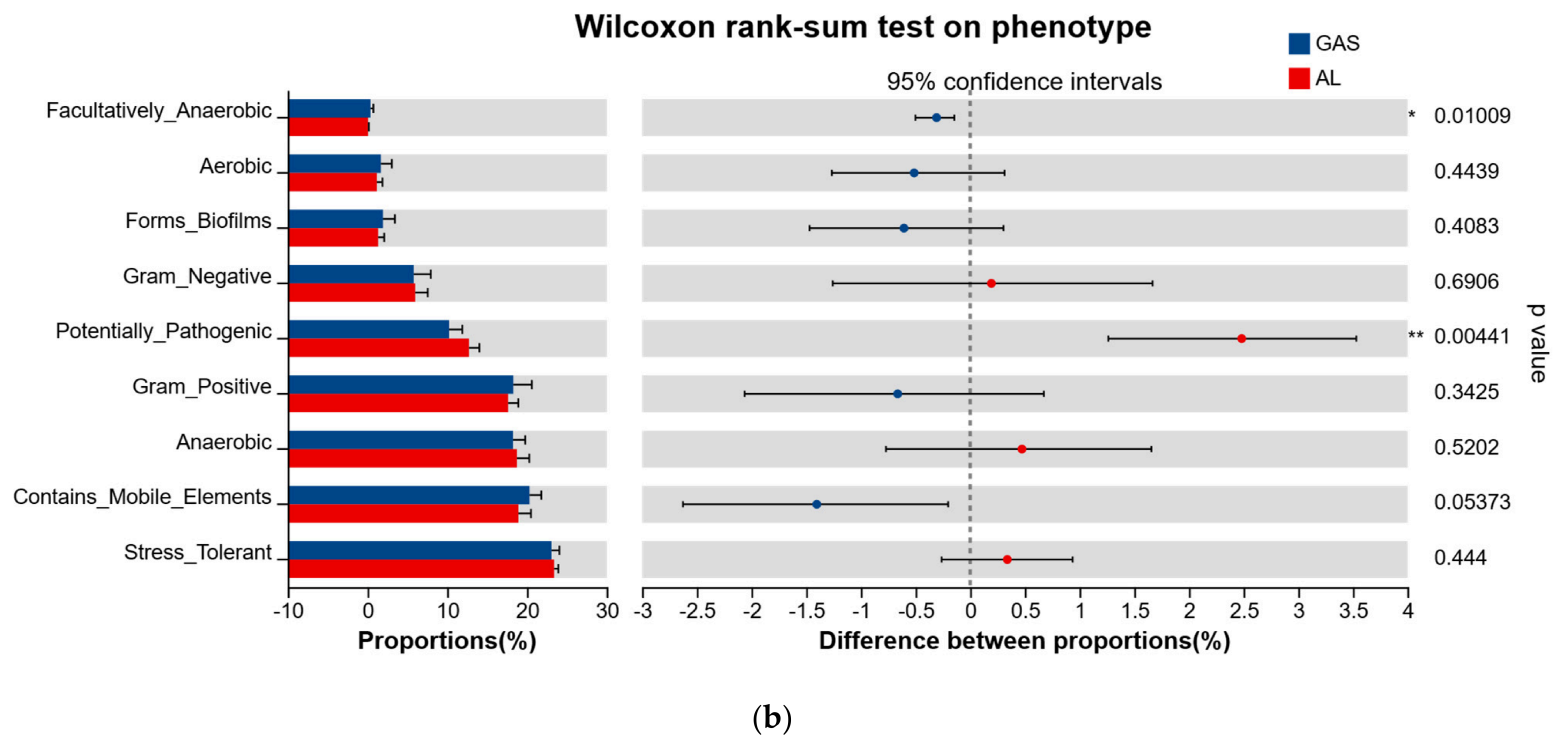
Predicted functional profiles of gut microbiota in guanacos (GAS) and alpacas (AL). (**a**) KEGG Level 3 pathways (only pathways with significant differences are shown; full list in Supplementary Table S8). (**b**) BugBase-predicted microbial phenotypes (relative abundance). Error bars represent SD. 0.01 < * *p* < 0.05, 0.001 < ** *p* ≤ 0.01, *** *p* ≤ 0.001. Note: All functional predictions are based on 16S rRNA gene data (PICRUSt2 and BugBase) and should be interpreted as inferred potentials, not direct measurements.

As shown in Figure 6b, additionally, the results demonstrated that the AL group had a higher predicted relative abundance of taxa classified as potentially pathogenic by BugBase compared with the GAS group (*p* < 0.05), and the AL group had a lower relative abundance of facultatively anaerobic compared with the GAS group (*p* < 0.05).

## 4. Discussion

In this study, we provide the first multi-dimensional comparison of the gut microbiota between captive guanacos and alpacas under strictly controlled environmental and dietary conditions. Our key findings reveal that, despite similar alpha diversity, the two species harbor significantly distinct gut microbial communities (beta diversity). Notably, guanacos exhibited higher community stability (lower inter-individual variability and lower AVDs), a broader niche, and a higher positive edge ratio and modularity compared to alpacas, which showed a higher negative edge ratio and enrichment of potentially pathogenic functions. These results support the hypothesis that domestication is associated with a divergence in gut microbial diversity, stability, and functional potential between guanacos and alpacas and sheds light on how host evolutionary history—particularly domestication—may shape gut microbial ecology in South American camelids.

In this study, alpha diversity indices (Shannon, Sobs, and Shannoneven) showed no significant differences between GAS and AL groups, consistent with studies in other captive artiodactyls where closely related species under identical husbandry conditions exhibit comparable within-community diversity [19]. This suggests that the standardized diet and stable zoo environment provide sufficient resources to support similar microbial richness in both species.

In contrast, beta diversity analysis revealed significant separation between the two groups, a pattern interpreted as evidence of host-specific microbial assembly [19]. Because both species consumed identical diets and shared the same enclosure, the observed compositional differences are likely associated with host genetics, physiology, or evolutionary history rather than external factors. This interpretation aligns with a recent study showing that host phylogeny remains a primary determinant of gut microbiota structure even under uniform husbandry conditions [19]. The greater number of unique ASVs (2810) and genera (49) in GASs compared to ALs (1497 unique ASVs; 16 unique genera) indicates that guanacos have a more species-specific microbial signature, possibly reflecting their evolution in diverse, resource-limited wild habitats, whereas alpacas have undergone centuries of domestication under more uniform conditions [3].

Both the AVD and within-group Bray–Curtis dissimilarity were lower in GASs than in ALs, indicating that the guanaco gut microbiota is more stable (less variable among individuals) [20,21]. Niche breadth analysis showed that GASs had a significantly broader habitat niche than ALs. A broader niche reflects a higher proportion of generalist taxa, which exhibit greater metabolic plasticity and lower environmental sensitivity [22]. In contrast, the narrower niche in ALs suggests a specialist-enriched community more vulnerable to perturbations [23], consistent with their higher AVDs. Niche overlap was significantly higher in ALs than in GASs, intensifying competitive interactions among taxa. This interpretation is supported by the co-occurrence network results, where ALs had a substantially higher proportion of negative edges (37.8%) than GASs (18.9%).

In the co-occurrence network analysis, the GAS network contained 32 nodes and 37 edges, with a high proportion of positive edges (81.1%), whereas the AL network had 43 nodes and 90 edges, with a significantly lower proportion of positive edges. Positive edges in co-occurrence networks are generally interpreted as indicating potential syntrophic or shared habitat preferences among taxa [24], suggesting a highly cooperative community in the GAS microbiota that may contribute to its higher stability. The high modularity of the GAS network (0.691 vs. 0.534 in ALs) further supports a more compartmentalized and robust structure [25,26], as high modularity restricts disturbance propagation [21,26].

Negative edges, associated with niche divergence, resource overlap, or mutual exclusion [26], were nearly twice as frequent in ALs (37.8%) than in GASs (18.9%), consistent with the narrower breadth and higher overlap observed in the AL niche. Although negative interactions can sometimes enhance stability [27], the AL network’s lower modularity and higher average degree (4.19 vs. 2.31) make it more densely connected and potentially more vulnerable to perturbation [27,28]. Collectively, these network-level differences support a convergent picture: GASs have a cooperative, modular, stable network typical of generalist-dominated ecosystems, while ALs exhibit a more competitive, dense, less modular network characteristic of a specialist-inclined community.

Both groups were dominated by *Bacillota*, *Bacteroidota*, and *Verrucomicrobiota* (>97%), consistent with previous camelid studies [29]. The relative abundances of these major phyla did not differ substantially, suggesting conservation of the core structural framework.

At the genus level, the GAS microbiota was enriched in *Monoglobus* and *Bacteroides*, while the AL microbiota was enriched in *Rikenellaceae_RC9_gut_group* and *Muribaculaceae*. *Monoglobus pectinilyticus* is a pectin-degrading specialist bacterium in the human colon, which possesses a highly specialized glycobiome for pectin degradation [30]. Its higher abundance in guanacos may reflect the adaptation to fibrous wild diets. *Rikenellaceae_RC9_gut_group* has been reported in other domesticated herbivores and is often associated with the fermentation of complex polysaccharides; this genus has been linked to lipid metabolism and fatty acid composition in pigs [31] and shows age-dependent variations in ruminants [32]. The functional implications of these differences warrant further investigation using metagenomic or metabolomic approaches. LEfSe analysis confirmed these patterns, with Actinomycetota enriched in GASs—a phylum known for producing bioactive secondary metabolites [33]—potentially contributing to its lower predicted pathogenicity.

Inferred functional predictions (PICRUSt2) revealed that 21 KEGG Level 3 pathways significantly different between the two groups. The AL microbiota was enriched in pathways *Staphylococcus aureus* infection and drug metabolism, while the GAS microbiota was enriched in pathways related to flavone and flavonol biosynthesis. The infection-related pathway enrichment in ALs aligns with BugBase predictions, showing higher potentially pathogenic taxa and fewer facultative anaerobes in ALs. These functional differences align with the AL network’s higher negative edge ratio, as a less stable, more competitive community may be more permissive to opportunistic pathogens [31,34]. The enrichment of flavone and flavonol biosynthesis pathways in GASs is also noteworthy, as flavonols and flavones are known to possess anti-inflammatory, antioxidant, and antimicrobial properties that may contribute to gut health and resilience [35].

The differential taxonomic and functional profiles suggest that domestication in alpacas may have been accompanied by a shift toward a gut ecosystem with a higher proportion of negative co-occurrences and lower stability, which could be more permissive of potentially pathogenic taxa based on these predictive models. This pattern mirrors findings in wild vs. domestic pigs [9,10,36]. Wild boars harbor higher abundances of beneficial bacteria such as *Bifidobacterium* and *Lactobacillus*, whereas domestic pigs show higher levels of potentially pathogenic genera [9,10]. Our results extend these observations to South American camelids and introduce network-level and stability metrics for assessing domestication-associated changes in the microbiome.

However, several limitations of this study should be acknowledged. First, all animals were housed in a single zoo, so the results may not be fully representative of other captive populations or wild animals. Second, the cross-sectional design does not permit the establishment of causal relationships; longitudinal studies would be required to determine whether the observed differences in stability and network structure persist over time or fluctuate with seasonal or physiological changes. Third, we did not measure short-chain fatty acids or other microbial metabolites, which would help link the predicted functional differences to actual metabolic outputs. Fourth, the functional predictions (PICRUSt2 and BugBase) are inferential and not validated by metagenomic or metabolomic data; thus, statements about “pathogenic potential” should be interpreted with caution. Fifth, the sample size was modest (16 guanacos vs. 8 alpacas) and imbalanced, which reduces statistical power and may affect the robustness of multivariate analyses (e.g., ANOSIM). The alpaca group contained only two males, which precluded any meaningful analysis of sex-related differences. Therefore, our findings should be interpreted conservatively and validated in larger, balanced cohorts. Sixth, our co-occurrence networks are based on correlation and do not directly reflect biological interactions (e.g., cooperation or competition); cautious interpretation is therefore warranted. Finally, the specific mechanisms underlying the observed differences in community stability and network architecture remain speculative; future work should incorporate host immune and physiological measurements, larger and sex-balanced cohorts, and metagenomic sequencing to elucidate the host–microbe interactions that drive these patterns.

## 5. Conclusions

In conclusion, this study provides the first comprehensive, multi-dimensional comparison of the gut microbiota between captive guanacos and alpacas. Despite similar alpha diversity, the two species harbor compositionally distinct gut microbial communities (beta diversity), with guanacos exhibiting higher community stability (lower inter-individual variability and lower AVDs), a broader niche, and a co-occurrence network characterized by a higher proportion of positive edges and higher modularity. In contrast, alpacas showed a higher proportion of negative edges and predicted enrichment of potentially pathogenic functions. These findings suggest that domestication may influence not only the taxonomic composition but also the stability, niche structure, and interaction network of the gut microbiota in South American camelids, thereby establishing a microbiome baseline for camelid conservation and health management.

## Data Availability

The 16S rRNA amplicon sequencing data produced during this research are publicly accessible in the NCBI database, cataloged under the BioProject ID PRJNA1458602.

## Supplementary Materials

The following supporting information can be downloaded at https://www.mdpi.com/article/10.3390/microorganisms14061325/s1. Table S1. Sequencing depth and quality metrics of 16S rRNA genes from gut microbiota. Table S2. Taxonomic analysis of the GAS and AL groups at the phylum level of the gut microbiota (relative abundance, %). Table S3. Taxonomic analysis of the GAS and AL groups at the genus level of the gut microbiota (relative abundance, %). Table S4. Pairwise propr values for all genera in the GAS group. Table S5. Pairwise propr values for all genera in the AL group. Table S6. Topological parameters of empirical and random networks for GAS and AL groups. Table S7. Complete list of differentially abundant taxa identified via LEfSe analysis between GAS and AL groups. Table S8. Function analysis of the GAS and AL groups at the KEGG level 3 of the gut microbiota (abundance).

## Abbreviations

The following abbreviations are used in this manuscript:

AL	Alpaca
GAS	Guanaco
ASV	Amplicon Sequence Variant
AVD	Average Variation Degree
KEGG	Kyoto Encyclopedia of Genes and Genomes
LDA	Linear Discriminant Analysis
LEfSe	Linear Discriminant Analysis Effect Size
PCA	Principal Component Analysis
Sobs	Observed Richness
