# Supplementary material for: Comparative Analysis of Gut Microbiome Diversity, Stability, and Predicted Function in Captive Guanacos (Lama guanicoe) and Alpacas (Vicugna pacos)
Source: Microorganisms. 2026 Jun 13;14(6):1325. doi: 10.3390/microorganisms14061325 (PMC13305035; doi:10.3390/microorganisms14061325)
Supplement: Supplementary file 1 [file microorganisms-14-01325-s001.zip › microorganisms-4320810-supplementary.pdf]

Supporting Information

Supplemental Table S1. Sequencing depth and quality metrics of 16S rRNA genes from gut microbiota.

Supplemental Table S2. Taxonomic analysis of the GAS and AL groups at the phylum level of the gut microbiota (relative abundance, %).

Supplemental Table S3. Taxonomic analysis of the GAS and AL groups at the genus level of the gut microbiota (relative abundance, %).

Supplemental Table S6. Topological parameters of empirical and random networks for GAS and AL, including node count, edge count, average degree, clustering coefficient, modularity, and number of communities.

Supplemental Table S7. Complete list of differentially abundant taxa identified via LEfSe analysis between GAS and AL groups.

Supplemental Table S8. Function analysis of the GAS and AL groups at the KEGG level 3 of the gut microbiota (abundance).

Supplemental Table S1. Sequencing depth and quality metrics of 16S rRNA genes from gut microbiota.

Sample ID	Total read bases (bp)	Clean reads	Mean length	Min length	Max length	Goods coverage
AL1	18573338	45128	411.57	201	540	1
AL2	19751109	48134	410.34	231	540	0.999945
AL3	18838126	45688	412.32	259	524	0.999945
AL4	18091910	44027	410.93	278	514	1
AL5	19995502	48488	412.38	224	522	1
AL6	19743390	47965	411.62	202	529	0.999973
AL7	20914339	50861	411.21	232	540	1
AL8	16847794	40860	412.33	262	528	1
GAS1	18918688	45970	411.54	232	540	0.999945
GAS2	19274224	46802	411.82	201	539	1
GAS3	16729272	40587	412.18	262	522	0.999973
GAS4	18035195	43749	412.24	230	528	1
GAS5	19566540	47545	411.54	201	529	1
GAS6	17192337	41841	410.90	213	522	1
GAS7	17869678	43309	412.61	228	540	1
GAS8	15020517	36496	411.57	203	527	1
GAS9	18123246	43934	412.51	231	522	1
GAS10	21094212	51353	410.77	231	527	0.999945
GAS11	18386713	44686	411.46	235	528	0.999973
GAS12	19670582	47815	411.39	219	531	0.999973
GAS13	19517899	47515	410.77	261	540	1
GAS14	19143770	46465	412.00	231	540	0.999945
GAS15	17024051	41372	411.49	257	522	1
GAS16	16805227	40777	412.13	203	528	1
Summation	445127659	1081367				
Average	18546986	45057	411.65	230.29	530.08	0.999984042

GAS represents the Guanacos and AL represents the Alpacas.

Supplemental Table S2. Taxonomic analysis of the GAS and AL groups at the phylum level of the gut microbiota (relative abundance, %).

Species name	AL-mean (%)	AL-sd (%)	GAS-mean (%)	GAS-sd (%)	Statistic (%)	*p*_value	Fold Change	*p*_adjust	Effect size
p__Bacillota	70.78	4.399	69.46	5.169	73	0.6027	1.019	0.9197	1.322
p__Bacteroidota	24.55	5.776	24.02	4.717	72	0.646	1.02206	0.9197	0.5344
p__Verrucomicrobiota	3.312	2.835	4.078	2.795	46	0.2839	0.81216	0.6812	-0.7656
p__Patescibacteria	0.2833	0.1792	0.8616	0.3939	8	0.00068	0.32881	0.00542	-0.5783
p__Actinomycetota	0.1048	0.08167	0.873	0.5379	3	0.00021	0.12005	0.00337	-0.7683
p__Spirochaetota	0.6114	0.671	0.2226	0.2903	87	0.1681	2.74663	0.5378	0.3887
p__Thermodesulfobacteriota	0.1565	0.1501	0.2944	0.2688	36.5	0.0981	0.53159	0.5232	-0.1379
p__Pseudomonadota	0.07467	0.09042	0.09111	0.08752	57	0.6898	0.81956	0.9197	-0.01644
p__Cyanobacteriota	0.03117	0.05853	0.03819	0.03004	40	0.1448	0.81618	0.5378	-0.00702
p__Fibrobacterota	0.05446	0.08001	0.01336	0.03373	77	0.3406	4.07635	0.6812	0.0411
p__unclassified_k__norank_d__Bacteria	0.02055	0.01997	0.02072	0.02115	66.5	0.9005	0.9918	1	-0.00017
p__SAR324_clade(Marine_group_B)	0.01267	0.02375	0.00788	0.015	67.5	0.8091	1.60828	0.9958	0.0048
p__Campylobacterota	0.00445	0.01259	0.01576	0.04324	63	0.9575	0.28255	1	-0.0113
p__Chloroflexota	0.00548	0.0155	0.00788	0.02374	64	1	0.69561	1	-0.0024

Supplemental Table S3. Taxonomic analysis of the GAS and AL groups at the genus level of the gut microbiota (relative abundance, %).

Species name	AL-mean (%)	AL-sd (%)	GAS-mean (%)	GAS-sd (%)	Statistic (%)	p_value	Fold Change	p_adjust	Effect size
g__UCG-005	11.41	1.898	10.01	1.794	93	0.08094	1.13986	0.3464	1.405
g__Christensenellaceae_R-7_group	10.69	2.308	9.145	2.209	88	0.1501	1.16894	0.4396	1.545
g__Rikenellaceae_RC9_gut_group	9.459	3.058	6.48	1.819	107	0.00925	1.45972	0.1042	2.979
g__norank_f__[Eubacterium]_coprostanoligenes_group	5.926	1.553	5.608	1.021	80	0.3425	1.0567	0.5639	0.3179
g__unclassified_f__Lachnospiraceae	5.848	0.931	5.394	1.557	80	0.3425	1.08417	0.5639	0.4535
g__Monoglobus	3.937	0.6182	5.243	1.206	16	0.00363	0.75091	0.06471	-1.306
g__norank_f__UCG-010	4.997	0.9426	3.551	1.041	111	0.00441	1.40721	0.06735	1.446
g__norank_o__Clostridia_UCG-014	4.129	1.959	3.235	1.635	82	0.2838	1.27635	0.5424	0.8936
g__Akkermansia	3.25	2.853	3.817	2.832	51.5	0.4623	0.85145	0.6749	-0.567
g__Bacteroides	2.498	1.008	3.594	1.101	30	0.04022	0.69505	0.2391	-1.097
g__Alistipes	3.039	0.7889	2.506	0.5753	90	0.1184	1.21269	0.3959	0.5322
g__norank_o__RF39	2.663	1.311	2.622	1.367	62.5	0.9512	1.01564	0.9947	0.04046
g__unclassified_f__Ruminococcaceae	2.079	0.7898	2.363	0.6467	45	0.2573	0.87981	0.5243	-0.2834
g__unclassified_c__Clostridia	2.089	0.3348	2.327	0.6984	48	0.3425	0.89772	0.5639	-0.2379
g__Prevotellaceae_UCG-003	1.547	0.9409	2.669	1.439	34	0.07084	0.57962	0.3094	-1.122
g__Prevotellaceae_UCG-004	1.63	1.065	1.89	0.6338	46	0.2839	0.86243	0.5424	-0.26
g__unclassified_f__Oscillospiraceae	1.442	0.6425	1.904	0.3127	37	0.1046	0.75735	0.3729	-0.4619
g__UCG-002	1.626	0.5032	1.385	0.3954	81	0.3123	1.17401	0.5639	0.2408
g__norank_f__Ruminococcaceae	1.106	0.4781	1.455	0.4308	36	0.09218	0.76014	0.3562	-0.3491
g__norank_f__Peptococcaceae	0.8717	0.2954	1.363	0.5008	26	0.02165	0.63955	0.1655	-0.4911
g__NK4A214_group	1.051	0.2413	1.153	0.2878	50.5	0.4256	0.91154	0.6552	-0.1014
g__norank_f__Muribaculaceae	1.507	0.9594	0.6922	0.474	106	0.01104	2.17712	0.1144	0.8151

g__UCG-009	1.123	0.2992	1.066	0.1868	70	0.7362	1.05347	0.8426	0.05708
g__Ruminococcus	1.2	0.698	0.8427	0.578	83	0.2571	1.42399	0.5243	0.3572
g__dgA-11_gut_group	0.6641	0.1738	1.175	0.6543	31	0.04657	0.56519	0.2547	-0.5105
g__Phascolarctobacterium	0.7843	0.504	0.9318	0.6365	58	0.7363	0.8417	0.8426	-0.1473
g__Family_XIII_AD3011_group	0.6638	0.162	0.9284	0.2873	24	0.01555	0.71499	0.128	-0.2646
g__norank_o__Bacteroidales	0.1654	0.1809	1.243	0.5282	2	0.00017	0.13307	0.01942	-1.078
g__unclassified_o__Oscillospirales	0.635	0.2968	0.7242	0.2465	41	0.1683	0.87683	0.4396	-0.08932
g__Romboutsia	0.5141	0.3003	0.7744	0.452	45.5	0.27	0.66387	0.5313	-0.2603
g__norank_f__Barnesiellaceae	0.6014	0.3334	0.6458	0.3813	58	0.7362	0.93125	0.8426	-0.04435
g__Candidatus_Saccharimonas	0.2833	0.1792	0.8616	0.3939	8	0.00068	0.32881	0.03005	-0.5783
g__Candidatus_Soleaferrea	0.5429	0.1637	0.5722	0.1779	60	0.8302	0.94879	0.9158	-0.02931
g__norank_f__p-2534-18B5_gut_group	0.3357	0.7811	0.7722	0.7454	29	0.03	0.43473	0.1994	-0.4366
g__Lachnospiraceae_UCG-010	0.4487	0.1515	0.5364	0.1966	51	0.4435	0.8365	0.6731	-0.0877
g__norank_f__Bacteroidales_RF16_group	0.7032	0.9067	0.2485	0.1684	96.5	0.04999	2.82978	0.2547	0.4546
g__unclassified_f__Prevotellaceae	0.6069	0.4569	0.3389	0.356	91	0.1046	1.79079	0.3729	0.2679
g__Prevotellaceae_UCG-001	0.5528	0.6594	0.373	0.2671	64	1	1.48204	1	0.1798
g__Oscillibacter	0.2843	0.203	0.5579	0.1654	21	0.00925	0.50959	0.1042	-0.2737
g__Treponema	0.61	0.668	0.2139	0.2779	87	0.1681	2.8518	0.4396	0.3961
g__Roseburia	0.2843	0.2527	0.3488	0.4037	64	1	0.81508	1	-0.06454
g__norank_f__F082	0.3613	0.5248	0.2374	0.2125	63	0.9756	1.5219	1	0.124
g__Longibaculum	0.3172	0.7932	0.2728	0.4384	50	0.356	1.16276	0.5815	0.04435
g__norank_f__M2PB4-65_termite_group	0.04555	0.1288	0.5143	0.5249	18.5	0.00423	0.08857	0.06735	-0.4687
g__Turicibacter	0.1897	0.1259	0.3081	0.1712	38	0.1183	0.61571	0.3959	-0.1184
g__Paeniclostridium	0.149	0.1325	0.2958	0.1655	28	0.02953	0.50372	0.1994	-0.1468
g__unclassified_f__Peptostreptococcaceae	0.1795	0.07629	0.2504	0.1281	36.5	0.09818	0.71685	0.3686	-0.0709
g__norank_f__p-251-o5	0.136	0.1119	0.2682	0.3018	49	0.3746	0.50708	0.5956	-0.1322

g__[Eubacterium]_siraeum_group	0.2771	0.182	0.1033	0.08001	107	0.00916	2.68248	0.1042	0.1738
g__Clostridium	0.1493	0.1383	0.2076	0.1776	55.5	0.6238	0.71917	0.7457	-0.05823
g__norank_o__Clostridia_vadinBB60_group	0.298	0.1926	0.05583	0.07272	116	0.00153	5.33763	0.04083	0.2421
g__Mogibacterium	0.08665	0.08455	0.2507	0.104	10	0.00104	0.34563	0.03192	-0.1641
g__Lachnospiraceae_NK3A20_group	0.1298	0.1083	0.2072	0.1471	44	0.2318	0.62645	0.4961	-0.07742
g__norank_o__WCHB1-41	0.06165	0.05946	0.2564	0.216	30	0.03979	0.24044	0.2391	-0.1947
g__Anaerorhabdus	0.137	0.09444	0.1348	0.1583	75	0.5197	1.01632	0.6749	0.00223
g__Cellulosilyticum	0.01199	0.02877	0.2475	0.5235	24	0.01203	0.04844	0.1144	-0.2354
g__unclassified_f__Eggerthellaceae	0.02843	0.03205	0.2264	0.3207	27.5	0.02628	0.12557	0.194	-0.198
g__norank_f__Anaerovoracaceae	0.08186	0.04144	0.1685	0.1424	28	0.02929	0.48582	0.1994	-0.08666
g__Dorea	0.08631	0.0496	0.163	0.0808	24	0.0155	0.52951	0.128	-0.07672
g__Parabacteroides	0.15	0.1483	0.09676	0.1338	79	0.3709	1.55023	0.5956	0.05326
g__Anaerovorax	0.09248	0.06734	0.1355	0.05201	37.5	0.1112	0.68251	0.3838	-0.04299
g__unclassified_f__Anaerovoracaceae	0.1219	0.07381	0.1039	0.07052	76.5	0.4621	1.17324	0.6749	0.01798
g__Defluviitaleaceae_UCG-011	0.09179	0.06069	0.1147	0.04142	52.5	0.5003	0.80026	0.6749	-0.02294
g__Lachnospiraceae_NK4A136_group	0.186	0.1628	0.01576	0.06302	101.5	0.0029	11.80203	0.05636	0.1702
g__Desulfovibrio	0.07295	0.05577	0.1236	0.1103	50	0.4044	0.59021	0.6317	-0.05069
g__Adlercreutzia	0	0	0.1961	0.2074	4	0.00018	0	0.01942	-0.1961
g__Breznakia	0.09282	0.09158	0.101	0.1924	76	0.4758	0.91901	0.6749	-0.00822
g__[Eubacterium]_ruminantium_group	0.1274	0.09875	0.05874	0.07092	90	0.1087	2.16888	0.3814	0.06867
g__Ruminiclostridium	0.1233	0.09249	0.06268	0.04918	87	0.1676	1.96713	0.4396	0.06062
g__[Eubacterium]_brachy_group	0.09487	0.05035	0.09076	0.04537	63	0.9755	1.04528	1	0.0041
g__Odoribacter	0.09385	0.05311	0.08545	0.06161	73	0.6023	1.0983	0.7328	0.00841
g__Mailhella	0.00617	0.01744	0.1658	0.2256	15.5	0.00228	0.03718	0.05332	-0.1596
g__Papillibacter	0.08665	0.06972	0.08306	0.05223	60	0.8289	1.04322	0.9158	0.0036
g__Acutalibacter	0.03699	0.03333	0.1308	0.0592	7	0.00054	0.2828	0.03005	-0.09384

g__Butyribacter	0.04795	0.04906	0.1159	0.08299	28.5	0.03075	0.41372	0.1994	-0.06799
g__Pseudoflavonifractor	0.1295	0.209	0.02586	0.07106	81	0.1548	5.00773	0.4396	0.1036
g__norank_f__Erysipelotrichaceae	0.02158	0.03498	0.1242	0.08481	14.5	0.00249	0.17375	0.05332	-0.1026
g__norank_f__Dysgonomonadaceae	0.08254	0.04057	0.05908	0.03428	94.5	0.06602	1.39709	0.3006	0.02347
g__Negativibacillus	0.0596	0.08785	0.081	0.07034	49.5	0.3814	0.7358	0.6001	-0.02142
g__Prevotellaceae_Ga6A1_group	0.1278	0.1753	0.01062	0.0185	95.5	0.03404	12.0339	0.2143	0.1171
g__Coprococcus	0.00617	0.01744	0.131	0.08865	10	0.00076	0.04706	0.03005	-0.1248
g__Pygmaiobacter	0.05206	0.04524	0.06919	0.05907	54.5	0.5796	0.75242	0.7128	-0.01712
g__Paludicola	0.07604	0.06551	0.04316	0.06041	86	0.1662	1.76182	0.4396	0.03287
g__norank_f__Carnobacteriaceae	0.09796	0.2771	0.02072	0.05923	61.5	0.8504	4.7278	0.9238	0.07722
g__norank_f__Christensenellaceae	0.04144	0.0392	0.07073	0.07245	51	0.4403	0.58589	0.673	-0.02928
g__Blautia	0.05754	0.07538	0.05275	0.07618	68	0.8109	1.09081	0.9085	0.00479
g__Streptococcus	0.00719	0.01486	0.1002	0.1509	23.5	0.01175	0.07179	0.1144	-0.09299
g__norank_f__PeH15	0.087	0.2461	0.01867	0.04459	61.5	0.8504	4.65988	0.9238	0.06834
g__norank_o__Izemoplasmatales	0.08563	0.0738	0.01867	0.03726	108	0.00508	4.5865	0.07249	0.06696
g__[Eubacterium]_ventriosum_group	0.02158	0.03915	0.08083	0.04848	19	0.00601	0.26698	0.08038	-0.05925
g__Saccharofermentans	0.02124	0.01668	0.0798	0.08322	37	0.103	0.26617	0.3729	-0.05857
g__norank_f__Eggerthellaceae	0.0137	0.02127	0.08631	0.1705	59	0.7514	0.15873	0.8553	-0.07261
g__Aeriscardovia	0.03014	0.03564	0.0685	0.06824	42.5	0.1927	0.44	0.4533	-0.03836
g__Xylanibacter	0.07775	0.1365	0.01678	0.04927	81	0.1548	4.63349	0.4396	0.06096
g__Berryella	0.00343	0.00772	0.08734	0.07894	10	0.00084	0.03921	0.03005	-0.08391
g__unclassified_f__Christensenellaceae	0.05309	0.02759	0.03716	0.03896	87	0.164	1.42869	0.4396	0.01593
g__Moryella	0.03185	0.04106	0.05703	0.04041	42	0.1797	0.55848	0.4396	-0.02518
g__Olsenella	0.00445	0.01259	0.08134	0.07975	27	0.01484	0.05475	0.128	-0.0769
g__norank_o__Bradymonadales	0.07227	0.13	0.00497	0.01987	84.5	0.05931	14.55296	0.2759	0.06729
g__Intestinimonas	0.04864	0.08134	0.0274	0.08266	78	0.2444	1.77518	0.5078	0.02123

g__Fibrobacter	0.05446	0.08001	0.01336	0.03373	77	0.3406	4.07635	0.5639	0.0411
g__Massiliomicrobiota	0.00856	0.014	0.05874	0.09888	60.5	0.8267	0.14578	0.9158	-0.05017
g__norank_o__Rickettsiales	0.02637	0.05515	0.04007	0.06015	42	0.1661	0.6581	0.4396	-0.0137
g__Acetitomaculum	0.03391	0.04643	0.03185	0.03356	59	0.7771	1.06468	0.8799	0.00206
g__Gordonibacter	0.01644	0.03131	0.04915	0.07081	49.5	0.3242	0.33449	0.5639	-0.03271
g__Family_XIII_UCG-001	0.02774	0.02099	0.03665	0.02487	51.5	0.46	0.75689	0.6749	-0.00891
g__Hydrogenoanaerobacterium	0.01918	0.01937	0.04127	0.03085	32.5	0.05451	0.46474	0.2651	-0.02209
g__Atopostipes	0.04521	0.1279	0.01387	0.04218	65	0.9575	3.25955	0.9947	0.03134
g__norank_o__Rhodospirillales	0.03288	0.0367	0.02552	0.03473	74	0.5403	1.2884	0.6762	0.00736
g__Oscillospira	0.02089	0.05909	0.02963	0.06788	59.5	0.706	0.70503	0.8212	-0.00874
g__norank_c__Clostridia	0.03733	0.05474	0.0113	0.01647	81	0.2706	3.30354	0.5313	0.02603
g__unclassified_o__Bacteroidales	0.02672	0.02351	0.02124	0.03199	73.5	0.5478	1.258	0.6816	0.00548
g__CAG-196	0.02911	0.0594	0.01764	0.02008	56.5	0.6465	1.65023	0.7686	0.01148
g__unclassified_p__Bacillota	0.01165	0.01687	0.03408	0.04323	47.5	0.295	0.34184	0.5586	-0.02243
g__unclassified_k__norank_d__Bacteria	0.02055	0.01997	0.02072	0.02115	66.5	0.9005	0.9918	0.9635	-0.00017
g__Atopobium	0.00274	0.00775	0.03716	0.03754	28	0.01778	0.07374	0.1409	-0.03442
g__[Eubacterium]_oxidoreducens_group	0.00925	0.02616	0.03031	0.03901	37	0.06985	0.30511	0.3094	-0.02106
g__Falcatimonas	0.01439	0.04069	0.02466	0.0627	57	0.5751	0.58354	0.7115	-0.01027
g__Lachnospiraceae_AC2044_group	0.02432	0.04052	0.01336	0.03717	78	0.2444	1.82036	0.5078	0.01097
g__Faecalibacterium	0.00993	0.01134	0.02517	0.01835	32	0.04924	0.39464	0.2547	-0.01524
g__unclassified_f__Butyricicoccaceae	0.00308	0.00872	0.02706	0.06037	50	0.2768	0.11393	0.5385	-0.02398
g__norank_f__Paludibacteraceae	0.01747	0.02172	0.01096	0.02208	77	0.3616	1.59398	0.5862	0.00651
g__unclassified_f__Rikenellaceae	0.00856	0.01229	0.01969	0.0216	48	0.3172	0.43489	0.5639	-0.01113
g__Marvinbryantia	0.01439	0.04069	0.01319	0.0314	61.5	0.8504	1.09098	0.9238	0.0012
g__Faecalitalea	0.00754	0.01463	0.01832	0.0733	75	0.2633	0.4113	0.5266	-0.01079
g__Caproiciproducens	0.01713	0.03254	0.00702	0.0103	62	0.9128	2.43982	0.9719	0.0101

g__Anaerofustis	0.01404	0.02823	0.00891	0.01746	61	0.8553	1.57664	0.9244	0.00514
g__unclassified_f__Oxalobacteraceae	0.01062	0.01634	0.01199	0.02411	69.5	0.7029	0.88574	0.8212	-0.00137
g__Corynebacterium	0.00411	0.01162	0.01661	0.04711	52.5	0.3757	0.24744	0.5956	-0.0125
g__norank_p__SAR324_clade(Marine_group_B)	0.01267	0.02375	0.00788	0.015	67.5	0.8091	1.60828	0.9085	0.00479
g__[Clostridium]_methylpentosum_group	0.00651	0.01309	0.01284	0.03246	62.5	0.9358	0.50685	0.9914	-0.00633
g__Aminicella	0.00206	0.00581	0.0161	0.02632	45	0.1583	0.12764	0.4396	-0.01404
g__Campylobacter	0.00445	0.01259	0.01182	0.03463	63	0.9575	0.37673	0.9947	-0.00737
g__Coprobacillus	0.00891	0.01662	0.00651	0.01823	78	0.2444	1.36832	0.5078	0.0024
g__Anaerobutyricum	0.00137	0.00388	0.01353	0.02069	40.5	0.0932	0.10126	0.3562	-0.01216
g__unclassified_f__Paludibacteraceae	0.0137	0.02929	0	0	80	0.0478	1	0.2547	0.0137
g__Flexilinea	0.00548	0.0155	0.00788	0.02374	64	1	0.69561	1	-0.0024
g__Anaeroplasma	0.01233	0.01932	0.00086	0.00343	85	0.0531	14.39916	0.2643	0.01147
g__norank_f__gir-aah93h0	0.0113	0.01808	0	0	88	0.0123	1	0.1144	0.0113
g__Frisingicoccus	0.00925	0.02616	0.00188	0.00754	68.5	0.6095	4.9087	0.7328	0.00736
g__norank_f__UCG-011	0.00206	0.00581	0.00873	0.03494	67.5	0.7017	0.23529	0.8212	-0.00668
g__unclassified_o__Erysipelotrichales	0.00754	0.01463	0.00257	0.01028	75	0.2633	2.93305	0.5266	0.00497
g__unclassified_f__Erysipelatoclostridiaceae	0.00445	0.00828	0.00531	0.01252	66.5	0.863	0.83876	0.9281	-0.00086
g__Peptococcus	0.00171	0.00484	0.00771	0.01405	54	0.4127	0.22229	0.64	-0.00599
g__norank_f__Gastranaerophilaceae	0.00206	0.00581	0.00702	0.01919	63	0.9575	0.29269	0.9947	-0.00497
g__norank_f__Atopobiaceae	0.00137	0.00388	0.00685	0.01591	55	0.4636	0.2	0.6749	-0.00548
g__norank_f__Ethanoligenenaceae	0.00274	0.00775	0.00445	0.01315	63.5	1	0.61532	1	-0.00171
g__norank_f__Oscillospiraceae	0.00685	0.01277	0	0	80	0.0478	1	0.2547	0.00685
g__Colidextribacter	0.00377	0.01066	0.00086	0.00343	68.5	0.6095	4.40033	0.7328	0.00291
g__norank_f__Erysipelatoclostridiaceae	0.00206	0.00581	0.0024	0.00663	63.5	1	0.85696	1	-0.00034
g__Lachnospiraceae_NC2004_group	0.00171	0.00484	0.00257	0.01028	67.5	0.7017	0.6668	0.8212	-0.00086
g__V9D2013_group	0.00377	0.00701	0	0	80	0.0478	1	0.2547	0.00377

g__GWE2-31-10	0.00137	0.00388	0.00034	0.00137	68.5	0.6095	4	0.7328	0.00103

Supplemental Table S6. Topological parameters of empirical and random networks for GAS and AL groups.

Network Type	Empirical Network								Random Network		
	Similarity threshold	Number of Nodes	Number of Edges	Average Degree	Average Path Length	Average Clustering Coefficient	Modularity	Number of Communities	Random Average Path Length	Random Average Clustering Coefficient	Random Modularity
GAS group											
Total Graph	0.6	32	37	2.312	3.413953	0.3984848	0.691	6	3.56±0.73	0.077±0.08	0.521±0.1
UCG-002	0.6	9	10	2.222	2.083333	0.3500000	0.320	3	1.96±0.46	0.211±0.21	0.289±0.16
Prevotellaceae_UCG-003	0.6	7	7	2.000	2.000000	0.3750000	0.214	3	1.767±0.58	0.318±0.32	0.228±0.23
norank_f___Eubacterium__coprostanoligenes_group	0.6	5	5	2.000	1.700000	0.5833333	0.220	2	1.502±0.5	0.321±0.32	0.133±0.16
Romboutsia	0.6	6	9	3.000	1.533333	0.7333333	0.123	2	1.426±0.23	0.595±0.6	0.069±0.09
norank_o__WCHB1-41	0.6	3	2	1.333	1.333333	0.0000000	-0.125	2	1.143±0.14	0.481±0.48	-0.142±0.08
norank_f__p-251-o5	0.6	2	1	1.000	1.000000	NaN	-0.500	2	1±0	NaN	-0.5±0
AL group											
Total Graph	0.6	43	90	4.186	3.224806	0.4423854	0.534	5	2.709±0.26	0.096±0.06	0.385±0.06

norank_f__Muribaculaceae	0.6	6	7	2.333	1.866667	0.5333333	0.204	2	1.615±0.35	0.441±0.44	0.131±0.13
Lachnospiraceae_UCG-010	0.6	6	5	1.667	2.066667	0.0000000	0.260	3	1.637±0.64	0.272±0.27	0.164±0.16
Romboutsia	0.6	11	23	4.182	1.727273	0.6841991	0.247	3	1.639±0.18	0.42±0.26	0.164±0.08
Candidatus_Soleaferrea	0.6	13	26	4.000	1.897436	0.6121212	0.226	3	1.878±0.26	0.328±0.18	0.236±0.1
Lachnospiraceae_NK3A20_group	0.6	7	10	2.857	1.666667	0.6222222	0.120	2	1.597±0.31	0.491±0.49	0.133±0.11

Supplemental Table S7. Complete list of differentially abundant taxa identified via LEfSe analysis between GAS and AL groups.

Species name	Group	Mean	LDA value	*P*_value
p__Bacteroidota.c__Bacteroidia.o__Bacteroidales.f__Rikenellaceae	AL	5.11958	4.20872	0.01693
p__Bacteroidota.c__Bacteroidia.o__Bacteroidales.f__Rikenellaceae.g__Rikenellaceae_RC9_gut_group	AL	4.97583	4.20423	0.00846
p__Bacillota.c__Clostridia.o__Oscillospirales.f__UCG-010.g__norank_f__UCG-010	AL	4.69872	3.85803	0.004
p__Bacillota.c__Clostridia.o__Oscillospirales.f__UCG-010	AL	4.69872	3.85803	0.004
p__Bacillota.c__Clostridia.o__Monoglobales	GAS	4.71957	3.81827	0.00329
p__Bacillota.c__Clostridia.o__Monoglobales.f__Monoglobaceae	GAS	4.71957	3.81827	0.00329
p__Bacillota.c__Clostridia.o__Monoglobales.f__Monoglobaceae.g__Monoglobus	GAS	4.71957	3.81827	0.00329
p__Bacteroidota.c__Bacteroidia.o__Bacteroidales.f__Bacteroidaceae.g__Bacteroides	GAS	4.55563	3.75572	0.03734
p__Bacteroidota.c__Bacteroidia.o__Bacteroidales.f__Bacteroidaceae	GAS	4.55563	3.75572	0.03734
p__Bacteroidota.c__Bacteroidia.o__Bacteroidales.f__norank_o__Bacteroidales.g__norank_o__Bacteroidales	GAS	4.09463	3.72539	0.00015
p__Bacteroidota.c__Bacteroidia.o__Bacteroidales.f__norank_o__Bacteroidales	GAS	4.09463	3.72539	0.00015
p__Bacillota.c__Clostridia.o__Peptostreptococcales-Tissierellales	GAS	4.486	3.65885	0.004
p__Bacteroidota.c__Bacteroidia.o__Bacteroidales.f__Muribaculaceae	AL	4.17822	3.65771	0.01011
p__Bacteroidota.c__Bacteroidia.o__Bacteroidales.f__Muribaculaceae.g__norank_f__Muribaculaceae	AL	4.17822	3.65771	0.01011
p__Actinomycetota	GAS	3.94103	3.55232	0.00019
p__Actinomycetota.c__Coriobacteriia.o__Coriobacteriales	GAS	3.88887	3.51534	0.00019
p__Actinomycetota.c__Coriobacteriia	GAS	3.88887	3.51534	0.00019
p__Patescibacteria.c__Saccharimonadia.o__Saccharimonadales.f__Saccharimonadaceae	GAS	3.93529	3.44926	0.00061
p__Patescibacteria.c__Saccharimonadia.o__Saccharimonadales	GAS	3.93529	3.44926	0.00061
p__Patescibacteria.c__Saccharimonadia.o__Saccharimonadales.f__Saccharimonadaceae.g__Candidatus_Saccharimonas	GAS	3.93529	3.44926	0.00061
p__Patescibacteria.c__Saccharimonadia	GAS	3.93529	3.44926	0.00061
p__Patescibacteria	GAS	3.93529	3.44926	0.00061
p__Bacillota.c__Clostridia.o__Peptostreptococcales-Tissierellales.f__Anaerovoracaceae	GAS	4.23834	3.44375	0.00329
p__Actinomycetota.c__Coriobacteriia.o__Coriobacteriales.f__Eggerthellaceae	GAS	3.80975	3.42885	0.00024

p__Bacteroidota.c__Bacteroidia.o__Bacteroidales.f__Rikenellaceae.g__dgA-11_gut_group	GAS	4.0699	3.42782	0.0433
p__Bacteroidota.c__Bacteroidia.o__Bacteroidales.f__p-2534-18B5_gut_group.g__norank_f__p-2534-18B5_gut_group	GAS	3.88771	3.39948	0.0277
p__Bacteroidota.c__Bacteroidia.o__Bacteroidales.f__p-2534-18B5_gut_group	GAS	3.88771	3.38856	0.0277
p__Bacillota.c__Clostridia.o__Peptococcales.f__Peptococcaceae	GAS	4.13683	3.38426	0.0169
p__Bacillota.c__Clostridia.o__Peptococcales	GAS	4.13683	3.38426	0.0169
p__Bacillota.c__Clostridia.o__Peptococcales.f__Peptococcaceae.g__norank_f__Peptococcaceae	GAS	4.13438	3.38009	0.01996
p__Bacteroidota.c__Bacteroidia.o__Bacteroidales.f__Bacteroidales_RF16_group.g__norank_f__Bacteroidales_RF16_group	AL	3.84705	3.37907	0.04999
p__Bacteroidota.c__Bacteroidia.o__Bacteroidales.f__Bacteroidales_RF16_group	AL	3.84705	3.37907	0.04999
p__Bacteroidota.c__Bacteroidia.o__Bacteroidales.f__M2PB4-65_termite_group	GAS	3.71119	3.36935	0.00383
p__Bacteroidota.c__Bacteroidia.o__Bacteroidales.f__M2PB4-65_termite_group.g__norank_f__M2PB4-65_termite_group	GAS	3.71119	3.36643	0.00383
p__Bacteroidota.c__Bacteroidia.o__Bacteroidales.f__Paludibacteraceae.g__unclassified_f__Paludibacteraceae	AL	2.13672	3.25994	0.04105
p__Bacillota.c__Clostridia.o__Peptostreptococcales-Tissierellales.f__Anaerovoracaceae.g__Family_XIII_AD3011_group	GAS	3.96771	3.15335	0.01428
p__Bacillota.c__Clostridia.o__Oscillospirales.f__Oscillospiraceae.g__Oscillibacter	GAS	3.74659	3.1437	0.00846
p__Bacillota.c__Clostridia.o__Clostridia_vadinBB60_group.f__norank_o__Clostridia_vadinBB60_group	AL	3.47418	3.10037	0.00137
p__Bacillota.c__Clostridia.o__Clostridia_vadinBB60_group	AL	3.47418	3.10037	0.00137
p__Bacillota.c__Clostridia.o__Clostridia_vadinBB60_group.f__norank_o__Clostridia_vadinBB60_group.g__norank_o__Clostridia_vadinBB60_group	AL	3.47418	3.10037	0.00137
p__Bacillota.c__Clostridia.o__Lachnospirales.f__Lachnospiraceae.g__Cellulosilyticum	GAS	3.3935	3.06565	0.01099
p__Bacillota.c__Clostridia.o__Oscillospirales.f__Oscillospiraceae.g__V9D2013_group	AL	1.57606	3.01022	0.04105
p__Thermodesulfobacteriota.c__Desulfovibrionia.o__Desulfovibrionales	GAS	3.46152	3.00638	0.0048
p__Thermodesulfobacteriota.c__Desulfovibrionia.o__Desulfovibrionales.f__Desulfovibrionaceae	GAS	3.46152	3.00638	0.0048
p__Thermodesulfobacteriota.c__Desulfovibrionia	GAS	3.46152	3.00638	0.0048
p__Actinomycetota.c__Coriobacteriia.o__Coriobacteriales.f__Eggerthellaceae.g__Adlercreutzia	GAS	3.29244	2.98999	0.00016
p__Actinomycetota.c__Coriobacteriia.o__Coriobacteriales.f__Eggerthellaceae.g__unclassified_f__Eggerthellaceae	GAS	3.35487	2.97775	0.02427
p__Verrucomicrobiota.c__Kiritimatiellia.o__WCHB1-41	GAS	3.40886	2.96029	0.03692
p__Verrucomicrobiota.c__Kiritimatiellia.o__WCHB1-41.f__norank_o__WCHB1-41.g__norank_o__WCHB1-41	GAS	3.40886	2.96029	0.03692

p__Verrucomicrobiota.c__Kiritimatiellia.o__WCHB1-41.f__norank_o__WCHB1-41	GAS	3.40886	2.96029	0.03692
p__Verrucomicrobiota.c__Kiritimatiellia	GAS	3.40886	2.96029	0.03692
p__Bacillota.c__Bacilli.o__Lactobacillales	GAS	3.32425	2.95394	0.02458
p__Bacillota.c__Clostridia.o__Oscillospirales.f__Ruminococcaceae.g__[Eubacterium]_siraeum_group	AL	3.44261	2.9119	0.00837
p__Thermodesulfobacteriota.c__Desulfovibrionia.o__Desulfovibrionales.f__Desulfovibrionaceae.g__Mailhella	GAS	3.21951	2.90007	0.00205
p__Bacillota.c__Clostridia.o__Lachnospirales.f__Lachnospiraceae.g__Lachnospiraceae_NK4A136_group	AL	3.26946	2.8999	0.00254
p__Bacillota.c__Clostridia.o__Peptostreptococcales-Tissierellales.f__Anaerovoracaceae.g__Mogibacterium	GAS	3.39918	2.89061	0.00094
p__Bacillota.c__Clostridia.o__Lachnospirales.f__Lachnospiraceae.g__Coprococcus	GAS	3.1173	2.84979	0.00068
p__Actinomycetota.c__Coriobacteriia.o__Coriobacteriales.f__Atopobiaceae	GAS	3.09815	2.84406	0.00017
p__Bacillota.c__Clostridia.o__Peptostreptococcales-Tissierellales.f__Peptostreptococcaceae.g__Paeniclostridium	GAS	3.47093	2.82702	0.02732
p__Bacteroidota.c__Bacteroidia.o__Bacteroidales.f__Prevotellaceae.g__Prevotellaceae_Ga6A1_group	AL	3.10637	2.80354	0.03126
p__Bacillota.c__Clostridia.o__Oscillospirales.f__Ruminococcaceae.g__Acutalibacter	GAS	3.11673	2.80246	0.00048
p__Bacillota.c__Clostridia.o__Oscillospirales.f__Oscillospiraceae.g__norank_f__Oscillospiraceae	AL	1.83569	2.76587	0.04105
p__Bacillota.c__Bacilli.o__Erysipelotrichales.f__Erysipelotrichaceae.g__norank_f__Erysipelotrichaceae	GAS	3.09397	2.72613	0.00225
p__Bacillota.c__Bacilli.o__Acholeplasmatales	AL	2.14745	2.68427	0.04252
p__Actinomycetota.c__Coriobacteriia.o__Coriobacteriales.f__Eggerthellaceae.g__Berryella	GAS	2.9412	2.66286	0.00075
p__Bacillota.c__Clostridia.o__Lachnospirales.f__Lachnospiraceae.g__Dorea	GAS	3.21227	2.66255	0.01424
p__Bacillota.c__Clostridia.o__Peptostreptococcales-Tissierellales.f__Anaerovoracaceae.g__norank_f__Anaerovoracaceae	GAS	3.22663	2.66078	0.02708
p__Actinomycetota.c__Coriobacteriia.o__Coriobacteriales.f__Atopobiaceae.g__Olsenella	GAS	2.91033	2.66003	0.01353
p__Bacillota.c__Bacilli.o__Acholeplasmatales.f__Acholeplasmataceae	AL	2.14745	2.641	0.04252
p__Bacillota.c__Bacilli.o__Lactobacillales.f__Streptococcaceae.g__Streptococcus	GAS	3.00079	2.63124	0.00981
p__Bacillota.c__Bacilli.o__Izemoplasmatales.f__norank_o__Izemoplasmatales.g__norank_o__Izemoplasmatales	AL	2.9326	2.63038	0.00461
p__Bacillota.c__Bacilli.o__Izemoplasmatales.f__norank_o__Izemoplasmatales	AL	2.9326	2.62972	0.00461
p__Bacillota.c__Bacilli.o__Izemoplasmatales	AL	2.9326	2.62968	0.00461
p__Bacillota.c__Bacilli.o__Lactobacillales.f__Streptococcaceae	GAS	3.00079	2.62765	0.00981
p__Actinomycetota.c__Coriobacteriia.o__Coriobacteriales.f__Atopobiaceae.g__Atopobium	GAS	2.57009	2.58669	0.01624

p__Bacillota.c__Clostridia.o__Lachnospirales.f__Lachnospiraceae.g__[Eubacterium]_ventriosum_group	GAS	2.90758	2.57086	0.00602
p__Bacteroidota.c__Bacteroidia.o__Bacteroidales.f__gir-aah93h0.g__norank_f__gir-aah93h0	AL	2.05318	2.56114	0.01057
p__Actinomycetota.c__Actinobacteria	GAS	2.99481	2.55926	0.04826
p__Bacillota.c__Bacilli.o__Acholeplasmatales.f__Acholeplasmataceae.g__Anaeroplasma	AL	2.09097	2.55489	0.04252
p__Bacillota.c__Clostridia.o__Lachnospirales.f__Lachnospiraceae.g__Butyribacter	GAS	3.06422	2.54637	0.02844
p__Bacteroidota.c__Bacteroidia.o__Bacteroidales.f__gir-aah93h0	AL	2.05318	2.54117	0.01057
p__Bacillota.c__Clostridia.o__Oscillospirales.f__Ruminococcaceae.g__Faecalibacterium	GAS	2.40095	2.49259	0.04574

Only taxa with LDA scores > 2.0 are included (total 82 taxa). Columns include taxonomic level, taxon name, LDA score, and enrichment group (GAS or AL).

Supplemental Table S8. Function analysis of the GAS and AL groups at the KEGG level 3 of the gut microbiota (abundance).

Name	Fold Change (GAS/AL)	GAS-Mean	GAS1-Sd	AL-Mean	AL-Sd	Lower ci	Upper ci	Effect size	*P*-value	corrected *P*-value
Staphylococcus aureus infection	0.494	1636	592.2	3314	953.7	-2303	-1075	-1678	0.0004775	0.04902
D-Arginine and D-ornithine metabolism	0.737	930.9	372.2	1263	302.6	-599.4	-22.42	-332.5	0.01878	0.4131
Bladder cancer	0.005	0.04	0	7.925	11.79	-13.86	-2.986	-7.885	0.0236	0.4276
Degradation of aromatic compounds	0.904	29190	2244	32280	2271	-4874	-1084	-3086	0.009216	0.258
Caprolactam degradation	0.457	149.3	89.51	326.9	149.7	-274.3	-85.39	-177.7	0.005198	0.2287
Naphthalene degradation	0.869	15480	925	17810	1511	-3295	-1422	-2321	0.001199	0.09235
Thyroid hormone signaling pathway	0.711	296.5	70.54	417.3	135.7	-196.9	-38.84	-120.8	0.01878	0.4131
Flavone and flavonol biosynthesis	1.753	225.4	107.3	128.6	60.08	25.89	188.6	96.78	0.02223	0.4276
Shigellosis	1.069	25790	1082	24120	1535	715.1	2670	1668	0.009216	0.258
Longevity regulating pathway	0.896	15810	1214	17640	2092	-3175	-499.2	-1838	0.03082	0.4996
Cell cycle - yeast	0.252	22.87	19.82	90.71	35.86	-90.99	-46.74	-67.84	0.000228	0.03519

									5	
Platinum drug resistance	1.060	30790	1378	29050	2139	314.3	3073	1741	0.02622	0.4487
PI3K-Akt signaling pathway	1.031	45390	1040	44010	1181	527.5	2263	1378	0.01325	0.3401
Drug metabolism - cytochrome P450	0.744	4817	985	6472	1140	-2540	-810	-1654	0.003484	0.2146
Toluene degradation	0.273	45.01	29.36	165.1	158.7	-205	-51.47	-120.1	0.009185	0.258
Glucagon signaling pathway	1.020	192900	2392	189100	5943	600	6919	3813	0.04897	0.5604
Insulin signaling pathway	1.036	58850	1354	56800	2542	553.8	3645	2049	0.04897	0.5604
Metabolism of xenobiotics by cytochrome P450	0.740	4711	1002	6368	1151	-2489	-798.1	-1657	0.006315	0.2431
Cell cycle	0.246	22.87	19.82	93.03	35.85	-91.67	-49.48	-70.16	0.0001771	0.03519
Central carbon metabolism in cancer	1.018	147500	1952	144900	4251	51.26	4895	2545	0.02223	0.4276
Retinol metabolism	0.741	4650	1013	6273	1116	-2531	-755.3	-1623	0.004263	0.2189
